# INTERPROFESSIONAL HEALTH CARE: INTEGRATING AUDIOLOGY AND GERIATRICS IN INTERNATIONAL SETTINGS

**DOI:** 10.1093/geroni/igad104.1468

**Published:** 2023-12-21

**Authors:** Michelle Arnold, Jennifer Deal, Kathleen Pichora-Fuller

## Abstract

Hearing is fundamental to communication and important for quality of life and overall health of older adults. Despite a potentially large role in how patients engage in healthcare, its importance is underrecognized in healthcare settings. Decreased patient-provider communication and poor treatment understanding may drive decreased satisfaction with healthcare services and avoidance of routine medical care, resulting in poorer health outcomes for older adults with hearing loss. Additionally, despite a high prevalence of hearing loss, less than 20% of those who could potentially benefit from hearing healthcare use hearing aids, potentially exacerbating sequalae of hearing loss in healthcare settings. Amidst growing recognition of the need for inter-professional healthcare collaboration, this session will focus on how audiologists and hearing specialists may partner with other healthcare providers (pharmacists, nurses, physicians) to address hearing needs in primary care, specialty care and hospital settings. We will include perspectives from the United Kingdom, the United States and Canada. We will describe factors that facilitate and impede communication between pharmacists and patients with hearing loss in the community pharmacy. We will also present results of a mixed-methods study from an interdisciplinary team (gerontology, public health, audiology, and otolaryngology/ear nose throat (ENT)) to inform primary care/ENT physicians about preferences for hearing treatment among Black women in the United States. Within a hospital setting, we will present how audiologist-nurse partnerships impact delirium. We will conclude with a discussion of how policy for hearing aid coverage impacts primary care providers recommendations for hearing healthcare in the United States.

